# Retinal complications of gout: a case report and review of the literature

**DOI:** 10.1186/s12886-018-0669-6

**Published:** 2018-01-19

**Authors:** Ying Jiang, Jason E. Brenner, William J. Foster

**Affiliations:** 1 Department of Ophthalmology, Temple University, 3401 N Broad Street, 6th Floor Parkinson Pavilion, Philadelphia, PA 19140 USA; 2 Department of Bioengineering, Temple University, 3401 N Broad Street, 6th Floor Parkinson Pavilion, Philadelphia, PA 19140 USA

**Keywords:** Crystalline retinopathy, Gout, Hyperuricemia, Retina, Retinopathy, Uric acid, Case report

## Abstract

**Background:**

There have been few reported findings of posterior segment complications of gout. While exudative lesions, an increased risk of macular degeneration, and vascular occlusions have been previously reported, to our knowledge, refractile macular lesions have not been reported in a patient with chronic uncontrolled gout.

**Case Presentation:**

Highly refractile, crystal-like lesions were found in the macula of a 62 year old male patient with chronically uncontrolled gout. The lesions appeared at the termination of retinal arterioles and were located at the level of the retinal pigment epithelium. The lesions did not stain with fluorescein and were associated with larger areas geographic atrophy. Review of the patient’s blood tests revealed well-controlled vasculopathic risk factors. Fundus appearance and best-corrected visual acuity remained stable over 12 months of follow-up during which the uric acid levels were well controlled.

**Conclusion:**

Retinopathy may be associated with chronically uncontrolled gout and patients with visual complaints should undergo a dilated examination in addition to the typical anterior segment slit-lamp exam.

## Background

Gout is a systemic condition in which uric acid is deposited in tissues as monosodium urate, leading to an inflammatory reaction. The classic clinical finding is an inflammatory arthritis. However, the systemic nature of the condition has led to the involvement of various other organs, including the eye. Previous case reports and publications have demonstrated ocular involvement in the anterior segment. Several studies have noted associations with inflammatory reactions such as conjunctivitis and anterior uveitis [1–4]. There have also been reports of gouty crystal deposits in the cornea, sclera, and iris [5, 6]. A recent study by Lin et al. [7] investigated 380 patients with gout and reported two cases of corneal and one case of scleral uric crystal deposits. Additionally, these authors noted an association between gout and the presence of transparent conjunctival vesicles with metal-like reflection in the subconjunctival space, suggesting any similar findings warrant a suspicion of gout. Other associations between gout and elevated intraocular pressure, blurred disc margins, and possibly posterior uveitis have also been published in a case report [8].

To date, there have been few reported findings of posterior segment complications of gout. A case report [9] described an association between allopurinol use and exudative lesions in the macula. An older study in the French literature by Bourde [10] found that of 46 patients who presented with retinal vascular disorders without diabetes or hypertension, 76% had elevated uric acid levels. No retinal crystals were noted and the vascular findings were primarily venous occlusions and retinal hemorrhage. A more recent study [11] found an additional association between gout and Age-Related Macular Degeneration, which was hypothesized to be related to a systemic inflammatory reaction. However, there have not been any reports of direct urate crystal deposits in the retina. We describe here a patient with macular crystals and advanced systemic gout.

## Case report

**Fig. 1:**
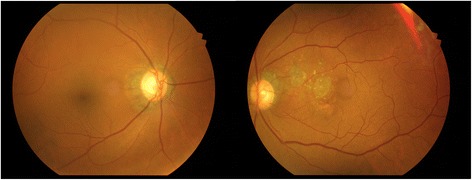
External photograph of the patient’s hands showing advanced gouty arthritis and tophi

**Fig. 2:**
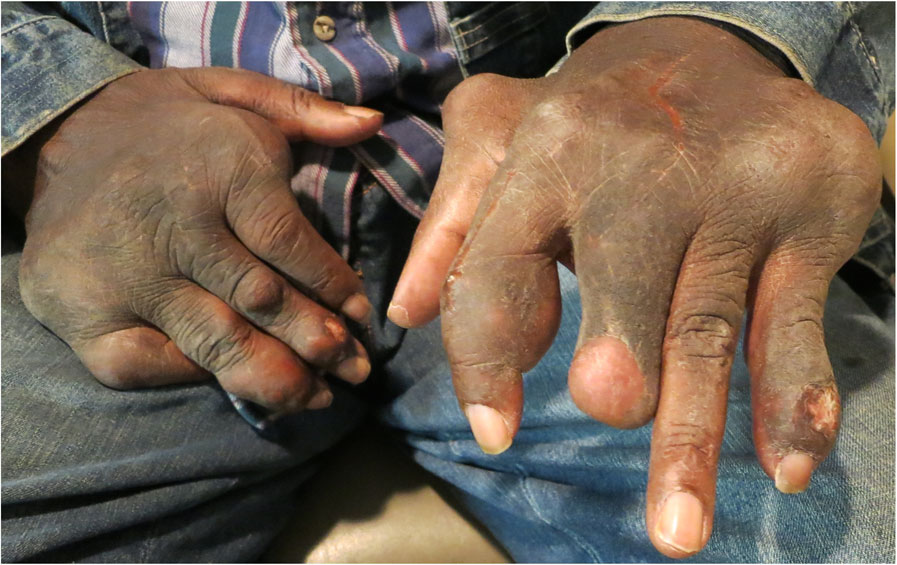
(Right) Red-free fundus of the left eye. The lesions can be seen to be predominantly at the termination of arterioles. (Left) Spectral domain OCT of the macula of the left eye demonstrating subretinal crystals

**Fig. 3:**
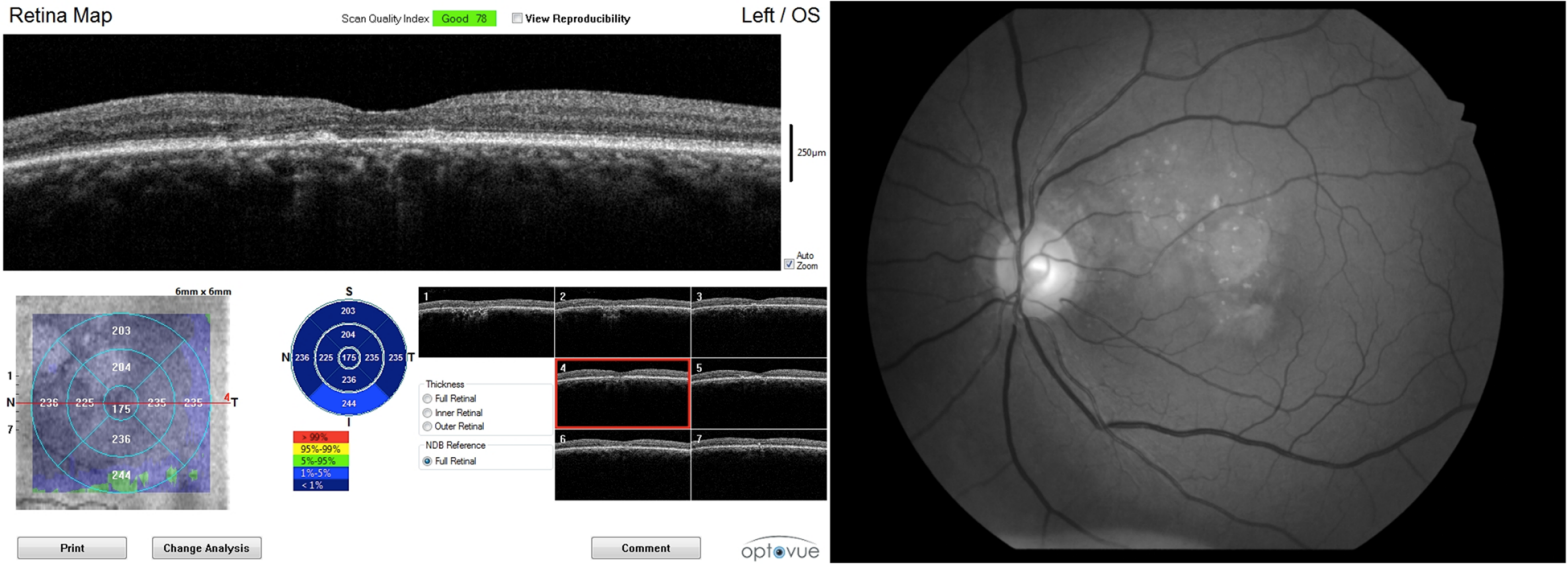
Fundus photographs demonstrating large areas of geographic atrophy and numerous smaller, refractile, yellow lesions in the left macula and RPE mottling in the right macula

A 62 year old African American male with a long-standing history of uncontrolled gout, demonstrated by chronic joint damage and tophi deposition in his hands (Fig. 1), presented with a complaint of slowly progressive blurred vision in both eyes and metamorphopsia in the left eye. His best-corrected visual acuity was 20/30 in both eyes. His anterior exam was normal except for mild bilateral nuclear and posterior subcapsular cataracts. He did not have any conjunctival or corneal pathology. His left macula demonstrated areas of geographic atrophy as well as subretinal, highly refractile lesions, which were predominately distributed at the termination of retinal arterioles in the macula, as can be seen in the red-free photo (Fig. 2, right). In comparison, the fundus exam in the right eye showed retinal pigment epithelium mottling (Fig. 3).

**Fig. 4:**
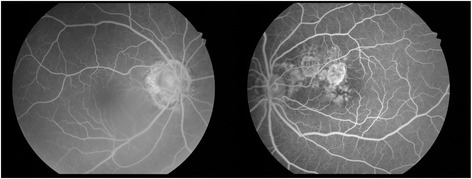
Fluorescein angiogram in the AV phase, demonstrating window defects and staining at the sites of atrophy and peripapillary atrophy. The refractile lesions do not stain

A fluorescein angiogram of the left eye (Fig. 4) demonstrated areas of atrophy with window defects and staining. The refractile lesions did not stain with fluorescein. The fluorescein angiogram of the right eye demonstrates peripapillary atrophy of the optic nerve (Fig. 4). Spectral-domain Ocular Coherence Tomography of the left eye (OCT; Optovue, Inc. Fremont CA) demonstrated subretinal lesions anterior to the Retinal Pigment Epithelium (RPE) (Fig. 2, left).

The patient was given an Amsler grid and followed in clinic. His retinal exam and visual acuity has been stable over the last 12 months, although he has reported a slight, subjective worsening of his metamorphopsia in the left eye.

Notably, the patient’s uric acid level was normal, at 5.3 mg/dL. The patient’s lipids have been well controlled (LDL < 145, Total Cholesterol <229) on a statin medication for several years, and he has no significant carotid artery or cardiac disease.

## Discussion

Retinal lesions have not previously been described in association with gout, which has most often been described as affecting the anterior segment including conjunctivitis, uveitis and corneal deposits.

The differential diagnosis of crystalline maculopathy is well-known and a common topic for board examinations. Commonly included in the list of possible (often rare) causes for this condition are: age-related macular degeneration, talc retinopathy, retinal emboli, tamoxifen retinopathy, canthaxanthin retinopathy, methoxyflurane retinopathy, hyperoxaluria, renal-related retinopathy (cilioretinopathies, Alport’s, cystinosis) retinitis punctata albescens, fleck retina of Kandori, fleck dystrophy, familial drusen, and Bietti crystalline dystrophy.

If we consider the most likely causes for the findings in this patient, it is unlikely that the findings described are related to hyperlipidemia and cholesterol emboli, in that the patient’s lipids have been well controlled on a statin medication for several years, and he has no significant carotid artery or cardiac disease. Additionally, retinal involvement in hyperlipidemia usually occurs in patients with familial hyperlipidemia syndromes and present at a much earlier age [12].

Age-related macular degeneration (ARMD) was also a consideration in this patient, however the lesions were inconsistent with drusen in that they were located anterior to the RPE and did not stain with fluorescein. There are no drusen visible on the basement membrane in either the photographs or the OCT. The distribution of the refractive crystals near the retinal vessels and their association with areas of atrophy is not consistent with non-exudative age-related macular degeneration. The crystals were also noted to be unilateral and the patient was of African American descent and macular degeneration is rare in this population, making ARMD unlikely. The uncommon genetic and toxic retinopathies are unlikely, as the patient does not have a history consistent with these conditions.

Although gout is a systemic disease, its typical presentation is often an asymmetric monoarticular joint involvement [13]. The disease’s predominantly asymmetric presentation correlates with previous cases of gout keratopathies that were primarily unilateral [4] and can also explain the primarily unilateral macular deposits in our patient.

Additionally, although the patient’s uric acid level was normal, at 5.3 mg/dL, at the time of presentation, it has been well documented that uric acid levels can be normal at the time of an attack of gout. A study [14] of 339 patients with gout demonstrated a serum level less than 8.0 mg/dL in up to 32% of patients with an acute flare. Furthermore, the patient had previously had poorly controlled gout, with a uric acid of 9.4 mg/dL. Thus serum uric acid cannot be used as an indicator of the chronicity of these macular lesions.

## Conclusion

In summary, we describe a patient with poorly controlled gout and macular lesions consistent with uric acid deposits or emboli. Patients with gout and visual symptoms should have a thorough examination and both the anterior and posterior segment should be carefully evaluated.

## Abbreviations

ARMD: Age-related macular degeneration
LDL: Low density lipoprotein
OCT: Ocular coherence tomography
RPE: Retinal pigment epithelium
