# Correction to: Retinal complications of gout: a case report and review of the literature

**DOI:** 10.1186/s12886-018-0691-8

**Published:** 2018-02-05

**Authors:** Ying Jiang, Jason E. Brenner, William J. Foster

**Affiliations:** 1 Department of Ophthalmology, Temple University, 3401 N Broad Street, 6th Floor Parkinson Pavilion, Philadelphia, PA 19140 USA; 2 Department of Bioengineering, Temple University, 3401 N Broad Street, 6th Floor Parkinson Pavilion, Philadelphia, PA 19140 USA

After publication of the article [1], it has been brought to our attention that the images displayed in Figs. 1, 2 and 3 have been transposed. Figure 1 should show the image from Fig. 2. Figure 2 should show the image from Fig. 3. Figure 3 should show the image from Fig. 1. The original article has now been revised to reflect this.
